# Suboptimal SVR rates in African patients with atypical genotype 1 subtypes: Implications for global elimination of hepatitis C

**DOI:** 10.1016/j.jhep.2019.07.025

**Published:** 2019-12

**Authors:** Kate Childs, Christopher Davis, Mary Cannon, Sarah Montague, Ana Filipe, Lily Tong, Peter Simmonds, Donald Smith, Emma C. Thomson, Geoff Dusheiko, Kosh Agarwal

**Affiliations:** 1 Institute of Liver Studies, King’s College Hospital Trust, United Kingdom; 2 MRC-University of Glasgow Centre for Virus Research, Glasgow, United Kingdom; 3 Nuffield Department of Medicine, University of Oxford, United Kingdom; 4 ICTV Online (10th) Report, University of Edinburgh, United Kingdom

**Keywords:** Hepatitis C, Antiviral Therapy, Africa, Treatment Failures

## Abstract

**Background & Aims:**

HCV subtypes which are unusual in Europe are more prevalent in the African region, but little is known of their response to direct-acting antivirals (DAAs). These include non-1a/1b/ non-subtypeable genotype 1 (G1) or non-4a/4d (G4). In this report we aimed to describe the genotype distribution and treatment outcome in a south London cohort of African patients.

**Methods:**

We identified all patients born in Africa who attended our clinic from 2010-2018. Information on HCV genotype, treatment regimen and outcome were obtained. Non-subtypeable samples were analysed using Glasgow NimbleGen next-generation sequencing (NGS). Phylogenetic analysis was carried out by generating an uncorrected nucleotide p-distance tree from the complete coding regions of our sequences.

**Results:**

Of 91 African patients, 47 (52%) were infected with an unusual subtype. Fourteen novel, as yet undesignated subtypes (G1*), were identified by NGS. Three individuals were infected with the same subtype, now designated as subtype 1p. Baseline sequences were available for 22 patients; 18/22 (82%) had baseline NS5A resistance-associated substitutions (RASs). Sustained virological response (SVR) was achieved in 56/63 (89%) overall, yet only in 21/28 (75%) of those with unusual G1 subtypes, with failure in 3/16 G1*, 1/2 G1p and 3/3 in G1l. Six treatment failures occurred with sofosbuvir/ledipasvir compared to 1 failure on a PI-based regimen. The SVR rate for all other genotypes and subtypes was 35/35 (100%).

**Conclusions:**

Most individuals in an unselected cohort of African patients were infected with an unusual genotype, including novel subtype 1p. The SVR rate of those with unusual G1 subtypes was 75%, raising concern about expansion of DAAs across Africa. Depending on the regimen used, higher failure rates in African cohorts could jeopardise HCV elimination.

**Lay summary:**

Direct-acting antiviral medications are able to cure hepatitis C in the majority of patients. The most common genotype of hepatitis C in Europe and the United States is genotype 1a or 1b and most clinical trials focused on these genotypes. We report that in a group of African patients, most of them had unusual (non-1a/1b) genotype 1 subtypes, and that the cure rate in these unusual genotypes was lower than in genotypes 1a and 1b.

## Highlights

• Unusual genotypes (G1 non 1a/1b or G4 non 4a/4d) were common in African patients.
• 11 previously unclassified HCV subtypes were represented including novel G1p.
• Patients with unusual G1 subtypes had a lower SVR rate than any other genotype.
• Failures were driven by patients treated with a first generation NS5A inhibitor.
• The majority of unusual G1 subtypes had baseline NS5A resistance mutations.

## Graphical abstract

**Figure:**
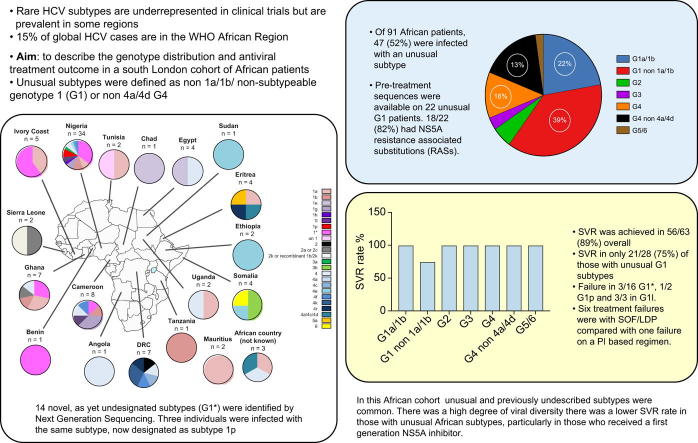

## Introduction

Direct-acting antiviral therapy (DAA) therapy has revolutionised hepatitis C (HCV) treatment. High cure rates with short courses of treatment make global eradication of HCV feasible; consequently, the World Health Organisation has promulgated a call for elimination of viral hepatitis as a public health threat by 2030.^1^

With 11 million people infected, HCV has an estimated prevalence of 1% in the African region.^1^ Despite this, there have been few clinical trials conducted in African cohorts and data is lacking on the prevalence, geographical distribution and treatment response of African sub-genotypes.[2], [3]

There are 8 known HCV genotypes which have been classified based on the analysis of HCV genetic sequences.^4^ Except for genotypes 5 and 8, each genotype is further divided into a number of subtypes. A genome-wide nucleotide sequence difference of 31–33% is considered sufficient to differentiate a genotype and a difference of 12–15% is sufficient to distinguish a sub-genotype (or subtype), although these boundaries are not strict and phylogeny is also considered.

Clinical trial and real-life data routinely report sustained virological response (SVR) rates in excess of 95% for genotype 1a, (G1a) 1b, 3 and 4. There are fewer data available on less prevalent genotypes such as 5 or 6, nor are unusual subtypes well represented. In an analysis of over 1,700 patients with genotype 1 HCV, who had been enrolled in clinical trials of NS5A inhibitors, less than 1% had subtypes of genotype 1 other than 1a or 1b.^5^ The available evidence suggests that other genotype 1 subtypes are common in Africa but less frequent in the industrialised countries where clinical trials have been centred.[6], [16] In this paper we refer to these as “unusual genotypes”. We choose this nomenclature for clarity as these genotypes are unusual in Europe, but not unusual in Africa, as we will discuss. However, there is a paucity of data as to whether the treatment response in patients with these less well characterised subtypes is comparable to more common subtypes. This has led to calls for more treatment outcome data in well characterised cohorts of patients.[7], [8]

Our institution serves a population of high ethnic diversity with a high prevalence of chronic hepatitis C infection. In this analysis we report the distribution of HCV genotypes and subtypes according to country of birth and treatment outcomes in an immigrant population cohort of patients born in Africa.

## Patients and methods

### Patients

This is a retrospective cohort study of all patients with hepatitis C at our centre who originate from the continent of Africa. We performed a search of our clinical database to identify all patients who were born in Africa, were infected with HCV and who accessed care between 2010 and 2018. For comparison, we also collected available information on the ethnicity and/or country of birth of all patients attending our clinic with HCV infection during the same time period. The study was approved by the Health Research Authority of England and Wales.

### Clinical data

We report on the genotypes, subtypes and treatment outcome in this group. The choice of hepatitis C treatment for all patients was discussed at a hepatitis multidisciplinary meeting and based on NHS England guidance. Data was gathered on the DAA regimen used including the use of ribavirin, the degree of hepatic fibrosis and the individuals’s HIV status. SVR was defined as an undetectable HCV RNA by sensitive polymerase chain reaction 12 and 24 weeks after cessation of treatment.

### Virological assays

HCV RNA was determined by the Roche COBAS® AmpliPrep/COBAS® TaqMan™ HCV Test, v2.0 assay with a sensitivity of 15 IU/ml.

For all patients, HCV genotyping was performed using the VERSANT HCV Geno 2.0 assay at our in-house tertiary viral hepatitis laboratory. Where samples could not be subtyped beyond belonging to genotype 1, they were designated ‘unassigned genotype 1’. These were sent to the MRC-University of Glasgow Centre for Virus Research, Glasgow for next-generation sequencing (NGS) with the aim of identifying the subtypes.

In this paper, we refer to all non-1a/1b/unassigned genotype 1 or non-4a/4d genotype 4 samples as “unusual African subtypes”. We use this term for want of a more precise label, as the epidemiological association with the region has been established. Many but not all of the identified sequences have been provisionally assigned genotypes; the majority of which have been given a notation based on sequences isolated from sub-Saharan, or North Africa. The word unusual refers to the low frequency with which these subtypes are seen in European treatment centres.

### Next-generation sequencing

NGS was carried out using a target enrichment sequencing protocol as previously described.^9^ Briefly, RNA was extracted from 200 μl plasma using the Agencourt RNAdvance Blood kit (Beckman Coulter) and reverse transcribed using SuperScript III (Invitrogen) with random hexamers and a NEB Second Strand Synthesis kit (New England Biolabs). Adapter-ligated DNA was amplified in real-time on an ABI 7500 cycler, using a KAPA Hifi Real-time library amplification kit. Index tags were added using NEBnext multiplex oligos (New England BioLabs). Pooled libraries were enriched using the NimbleGen SeqCap EZ system (Roche). Amplified DNA was purified using AMPure XP beads and eluted in a final volume of 15 μl. AnAgilent 2200 TapeStation was used to verify the final size profile of amplified library DNA. DNA libraries with appropriate index tags were pooled and paired end sequencing carried out on an Illumina MiSeq instrument using 300-cycle v2 reagents. *De novo* assembly was carried out using dipSPAdes and mapping with Tanoti (http://www.bioinformatics.cvr.ac.uk/tanoti.php). Sequence data were submitted to GenBank (accession numbers to follow).

### Phylogenetic analysis

An uncorrected nucleotide p-distance tree was generated using MEGA 7.0 from the complete coding regions of our sequences after alignment using MAFFT with a reference set of subtypes, including unassigned genotype 1 sequences. Maximum likelihood phylogenetic analysis was carried out using RaxML and the best fit model (GTR+G+I).

### Statistical analysis

Continuous variables were reported as median (interquartile range). Categorical univariate analysis was carried out using Chi Square and Fishers exact test. Analysis was performed using SPSS Statistics v25. When performing multivariate analysis, the small numbers led to issues of separation in the data therefore bias-reduced penalized-likelihood logistic regression (Firth 1993) was used.^10^ This was implemented in the 'logistf' R package and penalized-likelihood ratio tests were used to assess significance.^11^ Variables with a *p* value of <0.05 in univariate analysis were included in multivariate analysis.

## Results

### Sub-genotypes prevalent in African patients

**Fig. 1:**
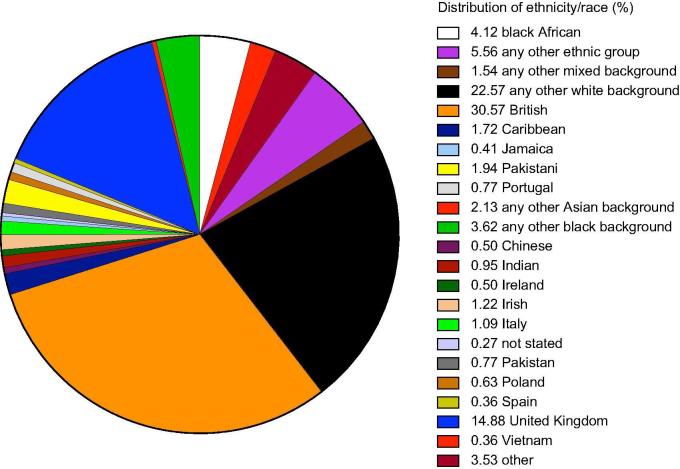
**Distribution of ethnicity/race of all patients (n = 2,211) with HCV attending our centre between 2010 and 2018.** (This figure appears in colour on the web.)

**Fig. 2:**
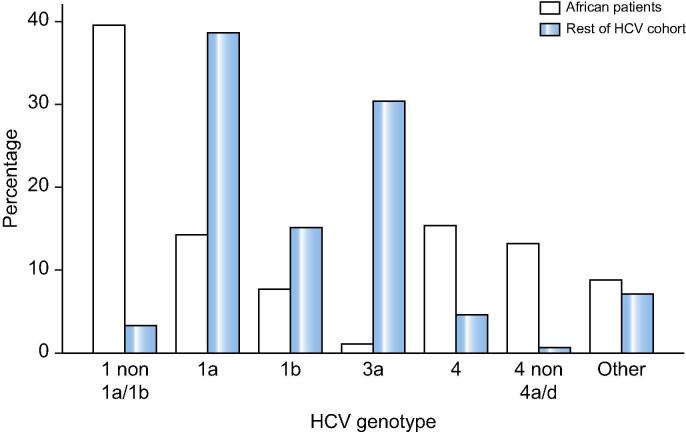
**Distribution of HCV genotype amongst 91 African patients (white bars) compared to the rest of the HCV cohort (blue bars).**

The total number of patients with HCV seen at our centre between 2010 and 2018 was 2,211, of whom we identified 91 (4.1%) patients who were born in Africa, the majority from sub-Saharan Africa (Table 1). The ethnicity or country of birth of the whole HCV cohort are shown in Fig. 1. Amongst the African patients 20/91 (22%) patients were infected with genotype 1a or 1b, 35 (39%) patients had unusual African genotype 1 subtypes, including sub-genotypes 1e, 1g, 1h, 1l, and 23 patients had unassigned genotype 1. Five patients (5.6%) were infected with genotype 2; 3 (3.3%) were infected with genotype 3; 14 (15.6%) had genotype 4; 12 (13.1%) were infected with unusual subtypes of genotype type 4, including 4c, 4e, 4f, 4k, 4r; 2 patients had genotype 5 and 6 infection. The frequency of HCV genotypes of the whole cohort compared to the African group are shown in Fig. 2. Of the non-African patients, who were predominantly British born, but also included Asian, Caribbean and other European backgrounds, 38.7% were infected with genotype 1a, 15.2% with G1b, 30.4% with G3a and only 3.3% with unassigned G1.

**Table 1:** **Demographics and clinical parameters. HCV genotypes are shown according to country of birth.**

**Parameter**	**N = 91**
Age	54 (47, 64)
Sex	42 (46%) female, 49 (54%) male
HIV positive	8 (9%)
Fibroscan (kPa) median (IQR)	6.1 (5.1, 8.7)
Cirrhosis	21/91 (23.1%)
Country of birth	n (genotype by country)
Angola	1 (4)
Benin	1 (1*)
Cameroon	8 (1*, 1b, 1e, 1e, 1e, 1l, 4, 4f)
Chad	1 (1e)
Democratic Republic of Congo	7 (2, 4, 4c, 4f, 4k, 4k, 4r)
Ivory Coast	5 (1a, 1a, 1*, 1*, 1*)
Egypt	4 (1g, 1g, 4, 4)
Eritrea	4 (1a, 4a/c/d, 4r, 5a)
Ethiopia	2 (4e, 4e)
Ghana	7 (1a, 1a, 1b, 1*, 1*, 2a/c, 4)
Mauritius	2 (1a, 1a)
Nigeria	34 (1a, 1a, 1a, 1*,1*, 1*, 1*, 1*, 1*, 1*, 1*, 1*, 1*, 1*, 1p, 1p, 1p, 1b, 1b, 1b, 1b, 1g, 1g, 1g, 1h, 1l, 1l, 3a, 4, 4, 4a/c/d, 4a, 4f)
Sierra Leone	2 (2k or 1b/2k, 2a or 2c)
Somalia	4 (3h, 3h, 4e, 6)
Sudan	1 (4e)
Tanzania	1 (1b)
Tunisia	2 (1a, 1*)
Uganda	2 (1a, 4)
African, country not known	3 (1a, 4, 4a/c/d)

**Fig. 3:**
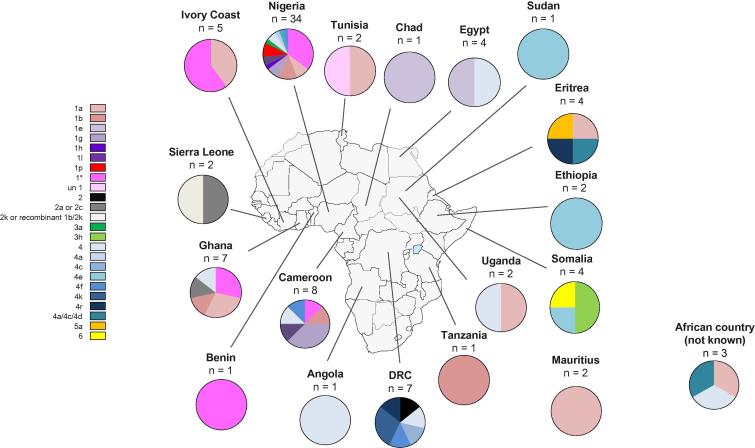
**HCV genotypes according to country of birth in African patients.** (This figure appears in colour on the web.)

In the African cohort, HCV genotypes according to country of birth are shown in Table 1 and Fig. 3. Of the 23 unassigned genotype 1 samples that underwent NGS, 18 complete open reading frame sequences from 15 novel unassigned subtypes were identified. Formal HCV sub-genotype assignment and classification requires at least 3 epidemiologically unrelated isolates.^4^ We met this recognised criterion with samples from 3 individuals from Nigeria, shown on the p-distance tree as Patient (P)15, P17, and P38. Two of the patients were a married couple, however phylogenetic analysis indicated that they were infected with different variants of the same subtype. This novel subtype has been assigned as 1p. It should be noted that 1 of our patients who failed treatment with sofosbuvir and ledipasvir, P17, was infected with the newly identified genotype 1p.

**Fig. 4:**
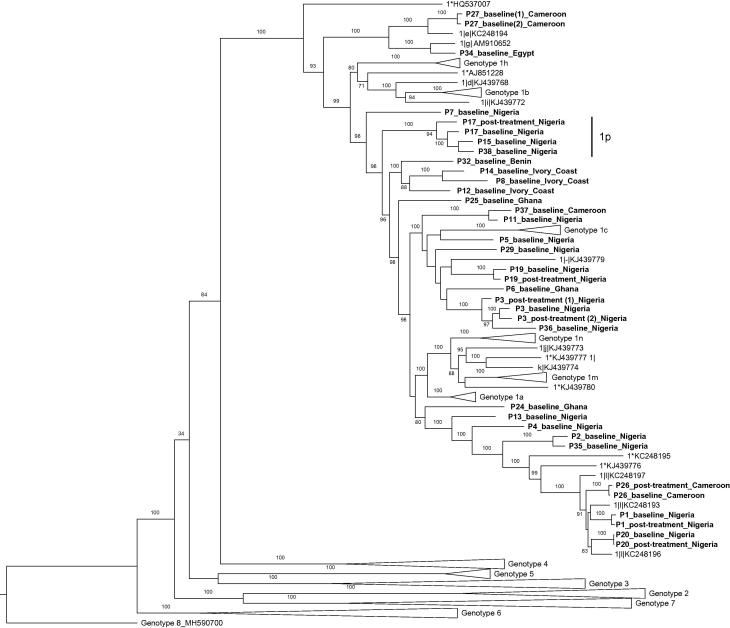
**Phylogenetic analysis of HCV complete genome sequences.** Our samples are in bold font and labelled by patient code and country of birth. Confirmed and previously reported unclassified reference sequences are shown in non-bold font. For patients who failed treatment, baseline and post treatment sequences are shown. P-distance corresponds to percentage of nucleotide positions at which sequence of interest differs from reference sequence and is measured horizontally. Novel subtype 1p is highlighted.

Of our remaining unassigned 14 subtypes, 3 had pairs of unrelated sequences and the remainder only 1 unrelated sequence. We refer to these novel unclassified subtypes as G1*. These are shown on the phylogenetic tree in Fig. 4.

### Treatment response

To date, 63 patients have completed DAA treatment and follow-up. Fifty-six of these patients have achieved an SVR, while 7 failed treatment, giving an overall SVR rate of 89%. As expected, response rates in genotypes 1a or 1b were uniformly high. Similarly, all patients infected with genotype 2, 3, 4 and 5 achieved an SVR. In those patients with unusual G1 and G4 African subtypes, treatment response according to genotype and choice of treatment regimen are shown in Table 2. An SVR was observed in only 21 of the 28 (75%) patients infected with unusual African genotype 1.

**Table 2:** **HCV treatment outcomes according to genotypes in African patients.**

**Genotype**	**Proportion treated**	**Treatment**	**SVR rate**	**SVR (NS5A based)**	**SVR (PI** **based)**
1a/1b			12/12 (100%)	2/2 (100%)	10/10 (100%)
1a and 1b	12/20	2 Sofosbuvir/ledipasvir 7 Paritaprevir/ritonavir/ombitasvir dasabuvir 3 Grazoprevir/elbasvir	2/2 7/7 3/3		
Distinct G1 subtypes			21/28 (75%)	9/15 (60%)	12/13 (92%)
1*	16/22	7 Sofosbuvir/ledipasvir	5/7		
		6 Paritaprevir/ritonavir/ombitasvir dasabuvir	6/6		
		3 Grazoprevir/elbasvir	2/3		
1e	2/4	1 Sofosbuvir/ledipasvir	1/1		
		1 Grazoprevir/elbasvir	1/1		
1g	5/5	3 Sofosbuvir/ledipasvir	3/3		
		1 Paritaprevir/ritonavir/ombitasvir dasabuvir 1 Grazoprevir/elbasvir	1/1 1/1		
1l	3/3	3 Sofosbuvir/ledipasvir	0/3		
1p	2/3	1 Sofosbuvir/ledipasvir 1 Paritaprevir/ritonavir/ombitasvir dasabuvir	0/1 1/1		
G4 subtypes			14/14 (100%)	2/2 (100%)	12/12 (100%)
Unknown 4	8/10	4 Paritaprevir/ritonavir/ombitasvir 4 Grazoprevir/elbasvir	8/8		
4e	3/4	1 Sofosbuvir/ledipasvir 1 Paritaprevir/ritonavir/ombitasvir, 1 Grazoprevir/elbasvir	3/3		
4f	2/3	1 Sofosbuvir/velpatasvir 1 Grazoprevir/elbasvir	2/2		
4k	1/2	1 Paritaprevir/ritonavir/ombitasvir	1/1		

PI, protease inhibitor; SVR, sustained virological response.

Five patients who failed initial treatment have thus far commenced retreatment: 3 of these patients have completed 16 weeks of glecaprevir and pibrentasvir, 2 of whom have achieved an SVR. One individual with cirrhosis and G1* HCV virus failed to achieve an SVR with 16 weeks of glecaprevir and pibrentasvir. Two other patients were retreated with sofosbuvir, velpatasvir and voxilaprevir and achieved an SVR.

### Factors associated with lack of SVR

A univariate categorical analysis was carried out to investigate which factors were associated with response. The presence of an unusual genotype 1 African subtype or the use of an NS5A inhibitor-based regimen were both significantly associated with a lack of SVR in univariate analysis, no other factors were. The results are shown in Table 3.

**Table 3:** **Factors associated with achieving an SVR.**

	**SVR**		
	**No**	**Yes**	***p* value by univariate analysis**
Genotype			
Unusual G1	7	21	0.002
Any other genotype	0	35	
Treatment regimen			
NS5a	6	16	0.008
Protease inhibitor based	1	40	
Cirrhosis			
No	4	46	0.135
Yes	3	10	
Ribavirin use			
No	4	23	0.6
Yes	3	33	
HIV positive			
No	6	49	1.0
Yes	1	6	

SVR, sustained virological response.

In multivariate analysis with SVR as the outcome variable, unusual genotype 1 remained highly significant with a *p* value of 0.016 (Chi-Squared: 5.78, df = 1), treatment regimen (NS5A inhibitor-based *vs*. protease inhibitor) was close to significant with a *p* value of 0.054 (Chi-Squared: 3.69, df = 1).

### HCV sequence results

Baseline sequence data was available for 22 patients, 14 who achieved SVR, 6 treatment failures, 2 who have not yet been treated. There were numerous NS5A polymorphisms present at baseline in patients with unusual G1 subtypes, particularly at positions 24, 30 and 31. Resistance-associated substitutions (RASs) listed in the EASL 2018 HCV guidelines as conferring reduced susceptibility to NS5A inhibitors or being associated with reduced treatment response were seen at baseline in 18/22 (82%) patients. All the treatment failures had either M28 polymorphisms (L and S) (2 failures) or L31M (4 failures). Five SVR patients had M28 (L and V), 3 patients (2 with 1* and 1 with 1g) who achieved SVR had Y93 (F/H/N) at baseline. These data are expanded in Table 4. The individual patient with 1* who has failed treatment with both sofosbuvir/ledipasvir and then glecaprevir/pibrentasvir had Q62E, L31M at baseline and Q62D, L31M following SOF/LDV. Following glecaprevir/pibrentasvir treatment Q30H, H58S and Y93H were also accumulated.

**Table 4:** **NS5A polymorphisms present at baseline and post treatment failure, where applicable.**

**SVR**	**Genotype**	**Baseline polymorphisms**	**Post-treatment RAS**
No	1l	**K24G**, **L31M,** H58P	**K24G**, M28L, **L31M,** H58P,
No	1l	K24S, **L31M,** H58P,	K24S, **L31M,** H58P,
No	1l	**K24G, Q30R**, **L31M**, H58P	**K24G**, **Q30R, L31M**, H58P
No	1*	M28L, H58P, **Q62D**	M28F, H58P, **Q62D,**
No	1p	M28S, **Q30L**, H58P, Q62P	M28S, **Q30L,** H58P, **Q62D**
No	1*	Q62E, **L31M,** H58P,	**Q62D, L31M**
Yes	1*	**Q62D,** H58P	n.a.
Yes	1g	**K24R**, M28L, H58P, **Y93F**	n.a.
Yes	1e	K24Q, M28L, **Q30R, L31M,** H58P, A92T	n.a.
Yes	1*	**K24G**, H58P	n.a.
Yes	1*	M28L, **Q30L, L31M,** H58P	n.a.
Yes	1*	**M28V**, H58P, **Q62D**	n.a.
Yes	1*	K24Q, Q62K, **Y93H,** H58P	n.a.
Yes	1*	S38A, H58P, **Q62D**	n.a.
Yes	1*	Q62E	n.a.
Yes	1*	**M28V, Q30T,** H58P, **Y93N**	n.a.
Yes	1*	H58P	n.a.
Yes	1*	K24Q, M28L, **Q30L**, H58P, Q62K, **L31M**	n.a.
Yes	1*	K24Q, M28L, **Q30R**, H58P,	n.a.
Yes	1*	H58P	n.a.
Untreated	1*	K24S, H58S,	n.a.
Untreated	1*	K24K, **L31M** H58P,	n.a.

G1 RASs as per 2018 EASL HCV Guidelines are in bold font. RAS, resistance-associated substitutions; SVR, sustained virological response.

## Discussion

In this analysis we report the prevalence of HCV genotypes in a consecutive cohort of 91 African patients from 18 countries, documenting the outcome of DAA therapy in patients with subtypes of genotype 1 and 4 that are prevalent in Africa but unusual in the West. Our study has 3 main findings. Firstly, unusual and previously undescribed subtypes were common. Secondly, there was a high degree of viral diversity and thirdly there was a lower SVR rate in those with unusual African subtypes, particularly in those who received a first generation NS5A inhibitor.

In this unselected cohort of immigrant African patients, 47 of 91 (52%) were infected by unusual African subtypes, (*i.e.* G1 non-1a/1b/ unassigned G1 subtype, or with G4 other than 4a or 4d). The distribution of genotypes was different from the non-African majority of our HCV patient cohort where G1a and 3a were the most common. The finding that unusual subtypes were common amongst African patients mirrors the results from the Los Alamos HCV database which contains 288 sequences of genotype 1c to 1m; 47.4% of these isolates originated in Africa.^12^ The fact the majority of catalogued non-1a/b G1 subtypes originated in Africa and that the majority of our cohort were infected with unusual subtypes suggests that these subtypes are more common in Africa than Europe. Since 15% of global cases of HCV are based in the WHO African region,^1^ these variants of HCV could be numerically significant. Population-based studies are required to establish the true prevalence of these “unusual” subtypes in Africa.

Our second finding was the high diversity of HCV sequences. We were able to sequence 15 previously undescribed subtypes of G1 HCV. One of these sequences was found to be a strain infecting 3 different patients, allowing formal classification as the novel genotype 1p. This is concordant with recent data from a cohort from Uganda and the Democratic Republic of Congo, where highly diverse genotype 4 and 7 strains, including unconfirmed novel strains were recently identified.^2^ We can speculate that the diversity of HCV observed in this cohort and other studies, if confirmed by larger phylogenetic analyses, perhaps points to an early evolutionary origin of human hepatitis C genotype 1 and 4 in Africa.^13^

We observed a high number of resistance-associated polymorphisms in pre-treatment samples from our cohort. NS5A RASs listed in the 2018 EASL guidelines as conferring reduced susceptibility to NS5A inhibitors, for example at positions 24, 28, 30 and 31 were present in 82% of patients with baseline sequence data.^14^ This contrasts with published findings from the pooled analysis of phase II and III studies of sofosbuvir and ledipasvir; of 2,108 patients only 16% patients had detectable baseline RASs.^15^ That we saw such high frequencies of polymorphisms further demonstrates the diversity of the virus in the African region.

Our third finding was that SVR rates were markedly lower in this population. Although we have studied a relatively small number of patients, we show that the overall SVR rate for African patients at an experienced centre (89%), is lower than expected in a group of predominantly non-cirrhotic patients. Importantly the SVR rate fell to 21/28 (75%), in those infected with non-1a/b G1 subtypes. The low SVR rate was driven by 6 failures in patients treated with sofosbuvir and ledipasvir, 1 failure with a protease inhibitor-based regimen and was associated with multiple baseline polymorphisms.

There is limited data available on treatment outcome in these uncommonly encountered genotypes. Zeuzem *et al.* have reported 25 patients with genotype 1 (1c/e/g/h/l) who all achieved SVR with an NS5a inhibitor-based regimen.^8^ These results were considered reassuring. It should be noted, however, that 18 patients received either sofosbuvir and velpatasvir, (7 with voxilaprevir) or 24 weeks of sofosbuvir and ledipasvir. Therefore, the majority of patients received the most potent treatment currently available, or else lengthened courses of treatment.

The French National Reference Centre recently reported that genotype 4r is associated with a lower response rate to DAAs. Five percent of all treatment failures were in patients infected with G4r, although this subtype is rare in the French population overall. Treatment failure was associated with multiple NS5A RASs which were present in all those analysed at baseline, similarly to our cohort.^16^ Gupta *et al*. recently published a large scale single arm trial in Rwanda showing that genotype 4r, present in 16% of participants, was associated with high rates of unsuccessful treatment with sofosbuvir and ledipasvir, despite good adherence.^17^ This has been linked to the presence of an amino acid motif, MPRMP, at positions 28-32 of the NS5A gene that confers high-level resistance to DAAs *in vitro*.^18^

Population-based studies to determine the frequency of unusual subtypes and the efficacy of DAA regimens against them, are urgently needed to formulate guidelines for treating HCV in Africa.^19^ The corollary of these data, and the fact that in resource poor regions of Africa genotyping or full genome sequencing will not be available, is that second generation NS5A inhibitors should be preferred as first-line agents in the region. Use of second-line NS5A inhibitors seems to overcome resistance.^20^ However further evaluation of the efficacy of newer regimens including sofosbuvir-velpatasvir or glecaprevir-pibrentasvir against the genotype 1 and 4 subtypes characterised in our analysis, is necessary. It has been suggested that a triple DAA regimen, including a protease inhibitor is required as first-line treatment.[16], [21]

We have shown that in a metropolitan United Kingdom cohort of African patients with HCV, most patients were infected with unusual and often novel African subtypes which were associated with reduced SVR rates. The reality of global migration means that these data are also relevant for clinicians in high income countries who should exercise caution in selecting regimens for African patients with unusual or un-subtypeable genotypes.

The evidence indicates that the future desired expansion of HCV treatment in Africa may risk unacceptable rates of failure if first generation NS5A inhibitors are utilised without appropriate epidemiological and viral sequence data. Global equity of access to curative treatment is required to avoid jeopardising the hepatitis C elimination agenda.

## Financial support

This work was supported by the Medical Research Council (MRC) (MC_UU_12014/1) and Wellcome (102789/Z/13/A) both to Emma Thomson.

## Conflict of interest

The authors declare no conflicts of interest that pertain to this work. Please refer to the accompanying ICMJE disclosure forms for further details.

## Authors’ contributions

Concept and design: K Childs, K Agarwal, M Cannon, G Dusheiko. Data collection: K Childs, S Montague. Whole genome sequencing and analysis, review of manuscript: EC Thomson, C Davis, A da Silva Filipe, L Tong. Phylogenetic analysis: D Smith, P Simmonds. Writing of article: K Childs, M Cannon, G Dusheiko, K Agarwal wrote the paper. All authors contributed to the preparation and review of the manuscript.
