# Supplementary material for: Suboptimal SVR rates in African patients with atypical genotype 1 subtypes: Implications for global elimination of hepatitis C
Source: J Hepatol. 2019 Dec;71(6):1099–105. doi: 10.1016/j.jhep.2019.07.025 (PMC7057256; doi:10.1016/j.jhep.2019.07.025)
Supplement: Supplementary Data 1 [file CTAT_table.pdf]

JOURNAL
OF HEPATOLOGY

Journal of Hepatology

CTAT methods

Tables for a "Complete, Transparent, Accurate and Timely account" (CTAT) are now mandatory for all revised submissions. The aim is to enhance the reproducibility of methods.

- Only include the parts relevant to your study
- Refer to the CTAT in the main text as 'Supplementary CTAT Table'
- Do not add subheadings
- Add as many rows as needed to include all information
- Only include one item per row

If the CTAT form is not relevant to your study, please outline the reasons why:

1.1 Antibodies

Name	Citation	Supplier	Cat no.	Clone no.

1.2 Cell lines

Name	Citation	Supplier	Cat no.	Passage no.	Authentication test method

1.3 Organisms

Name	Citation	Supplier	Strain	Sex	Age	Overall n number

1.4 Sequence based reagents

Name	Sequence	Supplier
1. Agencourt RNAdvance Blood Kit<br>2. SuperScript III<br>3. NEB Second Strand Synthesis Kit<br>4. Kappa library preparation kit<br>5. AMPureXP magnetic beads<br>6. NEBnext multiplex oligonucleotides<br>**7.** NimbleGen SeqCap EZ system		1. (Beckman Coulter)<br>2. (Invitrogen)<br>3. (New England Biolabs)<br>4. (KAPA BioSciences)<br>5. (Beckman Coulter)<br>6. (New England BioLabs)<br>7. (Roche).

JOURNAL
OF HEPATOLOGY

1.5 Biological samples

Description	Source	Identifier

1.6 Deposited data

Name of repository	Identifier	Link

1.7 Software

Software name	Manufacturer	Version
dipSPAdes: Assembler for Highly Polymorphic Diploid Genomes	Safonova, Y., Bankevic, A., Pevzner, P.A. (2014)	v1.0

1.8 Other (e.g. drugs, proteins, vectors etc.)

1.9 Please provide the details of the corresponding methods author for the manuscript:

Dr Emma Thompson, MRC-University of Glasgow Centre for Virus Research, Glasgow.

2.0 Please confirm for randomised controlled trials all versions of the clinical protocol are included in the submission. These will be published online as supplementary information.
